# Transvaginal Sacrospinous Ligament Fixation for Pelvic Organ Prolapse Stage III and Stage IV Uterovaginal and Vault Prolapse

**Published:** 2015-01

**Authors:** Pratiksha Gupta

**Affiliations:** Department of Gynecology and Obstetrics, Post Graduate Institute of Medical Sciences and Research, ESI Hospital, Basai Darapur, New Delhi, India

**Keywords:** Prolapse, Vaginal hysterectomy, Repair

## Abstract

The result of transvaginal sacrospinous ligament fixation technique, as part of the vaginal repair procedure for massive uterovaginal (Pelvic Organ Prolapse stage III and stage IV and vault prolapse) is evaluated. A total of 32 women were included in the present case series. *M*arked uterovaginal prolapse was present in 28 women and four had vault prolapse following hysterectomy. Patients with vault prolapse and marked uterovaginal prolapse underwent sacrospinous colpopexy. The mean follow-up period was 2.5 years. Out of the 28 patients with previous marked uterovaginal prolapse, only one had small cystocele 3 years after the surgery. This patient was asymptomatic and did not require repeat surgery. One woman had post-operative urinary tract infection and two had buttock discomfort, one had ischiorectal abscess and two had cuff cellulitis. All complications were dealt with successfully. No other major intra- and post-operative complications occurred. Transvaginal sacrospinous colpopexy can be performed together with vaginal hysterectomy, with marked uterovaginal prolapse and vault prolapse.

## Introduction

Sacrospinous colpopexy, introduced by Randall and Nichols ^1^ in 1971, has become a favored method for restoring vaginal support in women with vault prolapse, massive eversion of the vagina and procidentia***.*** Defects in apical vaginal support are crucial to recognize and address when undertaking surgery for prolapse. The upper third of the vagina (level I) is suspended from the pelvic walls by vertical fibers of the paracolpium, which is a continuation of the cardinal ligament.^2^ The uterosacral and sacrospinous ligament suspension seek to restore the level 1 vaginal support. The age-specific incidence increased with advancing age^3^ and thus better surgical techniques are required. In the present study, transvaginal sacrospinous ligament fixation technique was used as part of the vaginal repair procedure for marked uterovaginal prolapse and vault prolapse. Injury to the pudendal nerve, internal pudendal artery and vein, ureter and rectum is a possible complication.^4^ Exposure and direct visualization of the sacrospinous ligament coccygeus muscle complex require adequate dissection of the pararectal space, avoiding injury to the rectum. Injury to the pudendal nerve and the internal pudendal vessels is avoided by placing the fixation suture minimally 1.5 cm medial to the ischial spine. Vaginal prolapse is associated with weakness of pelvic floor due to childbirth and postmenopausal atrophy. The upper vagina is suspended in the pelvis by the caudal portion of the cardinal uterosacral ligament complex. These ligaments attach the cervix and upper vagina to the pelvic wall in the area of the greater sciatic foramen. When these suspensory fibers are damaged, the cervix, upper vagina prolapses downward away from the greater sciatic foramen, and fall below the normal position at the level of the ischial spine. Women who have undergone hysterectomy and in whom the suspensory apparatus was not reconstructed are at increased risk for vaginal eversion.^5^ The true incidence of vaginal vault prolapse following hysterectomy is approximately 0.5 percent of patients.^6^ Numerous operative techniques are described for the correction of vaginal prolapse.^7^ Fixation of the vaginal apex to the sacrospinous ligament has many advantages. By using a transvaginal approach, the incumbent potential complications of laparotomy are avoided and hospital stay as well as recovery to normal activity is shortened as well as maintenance of sexual potency.^8^ The purpose of this study was to evaluate if simple sacrospinous fixation, if done routinely with massive uterovaginal and vault prolapse, helps in long term follow up without any recurrence.

## Materials and Methods

A total of 32 women were included in the present case series. The study period was from January 2009 to January 2013 and carried out at the post-graduate Institute of Medical Sciences and Research, ESI Hospital (New Delhi, India). Prior to surgery, written informed consent was taken from all patients and approval from the Institutional Ethical Committee was obtained. Simple calculations were used for the interpretation of results. The inclusion criteria were women more than 40 years with pelvic organ quantification III and IV (POP-Q) stage, and vault prolapse after hysterectomy. Exclusion criteria were women more than 40 years with pelvic organ quantification I, II (POP-Q) stage, less than 40 years with III, IV (POP-Q) stage and women with serious medical disorder or not fit for anesthesia.

Pelvic organ prolapse was characterized and staged according to the International Continence Society Pelvic Organ Quantification (ICS POP-Q) staging system. Surgical procedures and outcome measures included anatomical and functional assessment of pelvic floor defects, according to POP-Q evaluation. Preoperatively, 27 had POP-Q stage IV and 5 had POP-Q stage III prolapse. 

All women were treated with sacrospinous ligament suspension of the vaginal vault. Twenty-eight women had marked uterovaginal prolapse, and four had vault prolapse following hysterectomy. The mean age of the patients was 58.5 years (range=42-75 years) and the mean parity was 5.5 (2-9). Eleven women had some systemic medical problems, but the operative procedure was not contraindicated in any of them after full evaluation. Out of four patients with vault prolapse, two had a previous vaginal hysterectomy and two had abdominal hysterectomy, which was done 3, 7, 4, 8 years back respectively. Out of the 28 patients with marked uterovaginal prolapse, 10 had previous pelvic surgeries. Six patients had cesarean sections among which four had cesarean section with ligation. Two had interval ligation; two had a vaginal Manchester repair done 6, 8 years back. Patients with marked uterovaginal prolapse underwent vaginal hysterectomy with obliteration of the enterocele sac, anterior and posterior vaginal repair if required, and sacrospinous colpopexy.

*Operative Technique*

**Figure 1:**
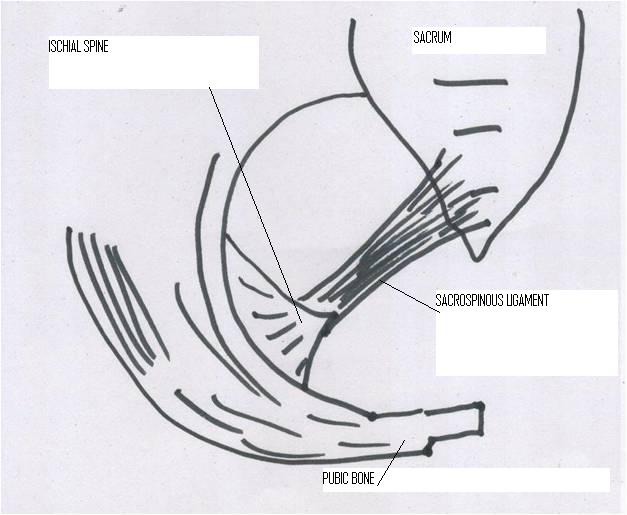
Description of the anatomy.

**Figure 2:**
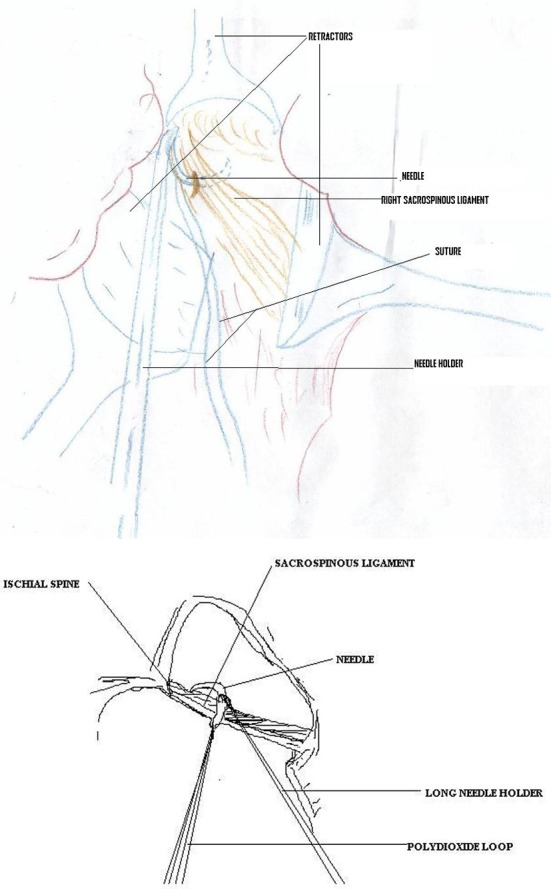
Presentation of the procedure.

Patients were operated under general anesthesia in lithotomy position. Vaginal hysterectomy was done as an initial step in patients of marked uterovaginal prolapse. For sacrospinous fixation, a longitudinal incision is given in the posterior vaginal wall to expose the rectovaginal space. The epithelium is dissected laterally and the pararectal space opens on the right side. The suspension is most often done to the patient’s right because retraction of the rectum is easier and a right-handed surgeon can pass a suture forehanded. By blunt finger dissection, a window is created between the rectovaginal space and ischial spine. If correctly identified, the plane will usually develop without any difficulty. Dissection is done until ischial spines are reached. Using the ischial spine as a prominent landmark, the sacrospinous ligament is palpated; this ligament passes from the ischial spine to the lower part of the sacrum. Now, three narrow malleable retractors are used to retract the peritoneum and rectum, to visualize clearly the sacrospinous ligament (figure 1-2). The upper border of the ligament will now be clearly defined. In all cases, a delayed absorbable suture 0 monofilament polydioxanone, 1.5 m (loop) on 40 mm half-circle, a heavy round body needle is used for this procedure. With a twelve inch long needle holder, the suture is placed through the sacrospinous ligament coccygeus muscle complex starting from the superior border in an upside down direction, it should be 2 cm medial to the ischial spine, so that neurovascular bundle is not injured. When this suture is retrieved, as it is a loop, now it is divided to establish two sutures. Thus, two suture pairs are established with one pass. After enterocele closure and anterior colporrhaphy (if indicated), sacrospinous sutures are placed through the full thickness of vaginal muscularis at the point of new vaginal apex. Vaginal cuff is now sutured and closed. The sutures of sacrospinous ligament are now tied. This tying of sutures brings the sacrospinous ligament in direct contact with the vaginal epithelium. When healing occurs, vaginal epithelium is fused with the sacrospinous ligament and vault remains suspended up nicely thereafter. Postoperatively women were given broad-spectrum antibiotic for five days.

## Results

Four women had vault repair with sacrospinous fixation and 28 had vaginal hysterectomy with sacrospinous fixation. Various surgical procedures done are shown in table 1. No major complications were reported. Immediate complications such as mild hemorrhage occurred in two women who responded well to compression during surgery. Cuff cellulitis developed in two women and UTI in one woman. All were treated with suitable antibiotic and the patients recovered. One woman had ischiorectal abscess and required abscess drainage through the vaginal route. The sacrospinous suture was removed through this vaginal approach. The patient was given intravenous broad-spectrum antibiotic for ten days and the patient responded to the treatment and abscess cavity healed within three weeks. Mild febrile morbidity was observed in four women with UTI, Cuff cellulitis and ischiorectal abscess. All responded well with antibiotics and no other major complications were reported. Follow-up examinations were performed annually on all patients. Out of the four patients with previous vault prolapse, none had recurrences. Out of the 28 patients with previous marked uterovaginal prolapse, only one had a small cystocele after 36 months following the operation. This patient was asymptomatic and did not require reoperation. Vaginal vault prolapse was not observed in any of these patients. Two women complained of dyspareunia after eight months, but in the following visit 24 months later, there were no further complaints (table 2).

**Table 1:** Various surgical procedure that were carried out

**Surgical procedure**	**Vault prolapse**	**POP-Q III**	**POP-Q IV**
Sacrospinous fixation	4	5	23
Anterior colporrhaphy	2	5	23
Enterocele repair	3	1	20
Bladder neck suspension	1	-	4

**Table 2:** List of complications

**Postoperative**	**No of cases**
UTI	1
Cuff cellulitis/infection	2
Febrile morbidity	-
Ischiorectal abscess	1
Nerve injury	-
Hematoma	-
Reoperation	-
Transfusion	-
**Recurrence**	
Cystocele	1
Enterocele	-
Total	5

## Discussion

There are different schemes to describe the extent of prolapse. The pelvic organ prolapse quantification (POP-Q) is now the most commonly used staging system. The quantitative assessment of the anterior, apical, and posterior vaginal supports, the genital hiatus, the perineal body, and vaginal length helps surgical planning when deciding upon the combinations of restorative transvaginal procedure, vaginal length and compartment-specific findings are important. An apical prolapse is more likely associated with a larger prolapse, 50% of cystocele size is explained by descent of apical support. The sacrospinous ligament suspension seeks to restore level I vaginal support. Pelvic floor dysfunction leads to surgery in 11% of women in their lifetime. A recurrence of prolapse occurs in approximately 25% within 5 years, the need for repeat surgery is 17%.^1^ In our study, only one woman had cystocele as recurrence which did not require any surgical intervention. The rates of anatomic failure in published studies vary significantly. Beer and Kuhn reviewed the literature and found that the failure rates ranged from 3% to 37%.^9^ The variation in outcome led to a systematic review to explain the differences in failure rates. Failure rates were higher in the anterior compartment and lower in the posterior and apical compartments. A meta-analysis illustrates that uterosacral ligament suspension is a highly effective procedure in restoring apical vaginal support. A successful anatomic outcome, defined as support of POP-Q stage 0 (“optimal”) or stage 1 (“satisfactory”), was reported in 98% of women. All studies, which met the inclusion criteria, were consistent in demonstrating this beneficial effect.^10^ In the present study, only one woman had a mild cystocele recurrence, which did not require any reoperation. The assessment of subjective symptoms is limited by the variation between studies on the outcome measures used. The relief from “vaginal pressure” or “bulge” symptoms were relieved by 82% to 100% of women.^2^ In the present study, this pressure symptom was relieved in 100% of women. Overall sexual function assessment, assessed by the Female Sexual Function Index (FSFI), 94% of the women were sexually active. Dyspareunia was relieved in 68% to 100% of patients who had it preoperatively and developed in 0% to 20.8% of patients who did not have it preoperatively.^6^ In our study, two women complained of dyspareunia at 8 months, but in the following visit at 24 months, there were no further complaints in our study group. Most common complications were febrile morbidity due to fever or abscess in 4.1% and hemorrhage and transfusion in 1.9% of patients. Two women had mild hemorrhage related to cystocele repair, which responded to compression. In a study by Nicholas,^4^ damage to the femoral and sciatic nerves were reported in 1.8%, gluteal pain, bladder pain, or unclassified pain in 0.8% and with the development of vaginal adhesions or rectovaginal fistula in 0.5% of patients respectively. None of our patients had any neurovascular injury. Only two women complained of nonspecific gluteal pain, which subsided after 5-6 days after surgery. In a study by Pasley,^11^ 94% percent of the patients who underwent sacrospinous suspension for uterovaginal prolapse and vaginal vault prolapse had no persistence or recurrence of vaginal vault prolapse 6 to 83 months after the procedure. This is similar to the cases in the present study. Our patients had the lowest complication rates compared with previous studies, which could be due to small sample size. Further studies are required with large sample size. Other surgeries have been classified and compared by different authors. In a study by Linda et al.,^12^ it was concluded that burch colposuspension significantly reduced postoperative symptoms of stress incontinence. In another study, 40 randomized controlled trials were carried out from which they concluded that abdominal sacral colpopexy was better than vaginal sacrospinous colpopexy in terms of a lower rate of recurrent vault prolapse and less dyspareunia.^13^^-^^15^

## Conclusion

Transvaginal sacrospinous colpopexy is a simple procedure and can be performed together with vaginal hysterectomy and vault prolapse. As abdominal sacropexy, this procedure is cheaper and is comparable with other procedures in terms of success rate. 
